# Salvage high-dose-rate interstitial brachytherapy for locally recurrent rectal cancer*

**DOI:** 10.1590/0100-3984.2013.1907

**Published:** 2016

**Authors:** Antônio Cássio Assis Pellizzon

**Affiliations:** 1 MD, PhD, Director of the Radiotherapy Department, A.C.Camargo Cancer Center, São Paulo, SP, Brazil.

**Keywords:** Rectal neoplasms, Salvage therapy, Brachytherapy, Radiotherapy

## Abstract

For tumors of the lower third of the rectum, the only safe surgical procedure is abdominal-perineal resection. High-dose-rate interstitial brachytherapy is a promising treatment for local recurrence of previously irradiated lower rectal cancer, due to the extremely high concentrated dose delivered to the tumor and the sparing of normal tissue, when compared with a course of external beam radiation therapy.

## Abstract (pt)

O procedimento cirúrgico padrão para tratamento dos tumores retais do terço distal é a ressecção abdominoperineal. A braquiterapia de alta taxa de dose é um tratamento promissor em tumores distais previamente irradiados, pela dose alta e extremamente concentrada de radiação administrada ao tumor e redução de dose aos tecidos normais se comparada à radioterapia externa.

## INTRODUCTION

Sphincter-sparing surgery and radiation therapy (RT) have made limited rectal tumors manageable. However, for tumors in the lower third of the rectum, abdominal perineal resection remains the only safe surgical procedure when loss of sphincter function is imminent. For locally advanced cancers, pre- or post-operative external RT is required and should be followed by surgery involving complete mesorectal excision^(1)^.

For localized cancers, two treatment strategies-surgery with or without adjuvant RT-can be proposed. In this situation, despite the scarcity of reports, the use of RT has been investigated as the sole treatment modality according to the characteristics of the tumor, as well as the condition, age, and life expectancy of the patient. Local control in rectal cancer is considered quite important because a relatively good prognosis is achieved in patients for whom local therapy is successful. After surgery, the expected rate of locally recurrent rectal cancer (LRRC) is 4-55%, depending on the pathological stage of the disease^(2-6)^.

The most common salvage treatment for LRRC after pre-or post-operative chemoradiotherapy (CRT) is abdominal perineal resection. Re-irradiation with a second external beam RT (EBRT) or CRT is considered, in general, for palliative treatment as less invasive therapy, because the total dose that can be given is limited by the first irradiation course. Therefore, high-dose-rate interstitial brachytherapy (HDR-IBT) is a promising treatment because the concentrated dose it delivers to the tumor is extremely high in comparison with that delivered by EBRT.

Literature on the management of local recurrence rates after RT for the lower third of the rectum is scarce. To our knowledge, there have been few reports on the use of HDRIBT for LRRC and none of those studies have involved long-term follow-up. Here, we present a case report and a review of the literature on the results of salvage HDR-IBT for LRRC.

## CASE REPORT

A 75-year-old female patient with controlled systemic hypertension presented with a history of nuclear grade II rectal adenocarcinoma (clinical stage of T1N0M0), treated with local resection and postoperative EBRT to a total dose of 54 Gy of 15 MeV photons in 30 fractions. The radiation treatment started in January 2000 and ended in February 2000. In May 2013, the patient presented with local pain and bleeding, using 2 g/day of ibuprofen for pain control.

Sigmoidoscopy showed the presence of a 2.5-cm ulcerated lesion in the left lateral to the anterior wall of the distal rectum, at 1.5 cm from the anal verge. The vaginal mucosa was not involved. The biopsy confirmed nuclear grade II adenocarcinoma, and magnetic resonance imaging showed a 30 mm × 25 mm mucosal enlargement. The patient refused surgical treatment of the LRRC and was referred to the Radiation Oncology Department for salvage treatment. Due to the high dose given to that area in 2000, the option was HDR-IBT.

**Figure 1:**
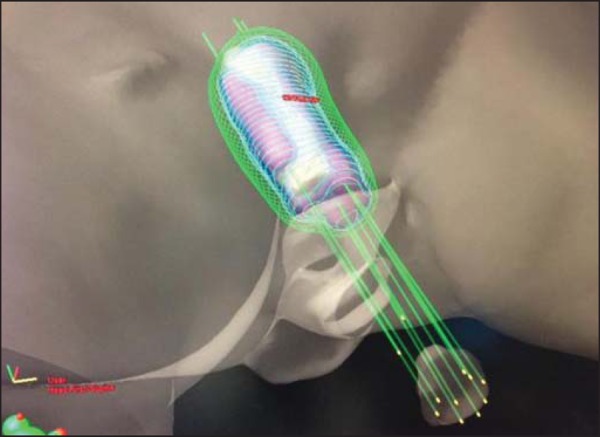
Three-dimensional view based on computed tomography images.

**Figure 2:**
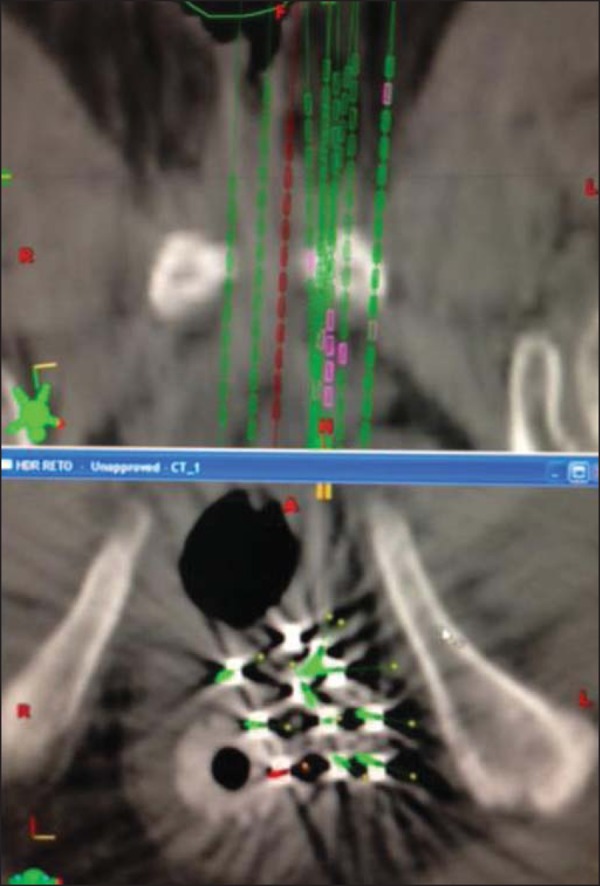
Planning computed tomography and reconstruction views in the axial and coronal planes.

In June 2013, the patient underwent an interstitial implant, which was performed in the operating room under spinal anesthesia. Eleven metallic needles were inserted through a perineal template, disposed in two layers but avoiding one fourths of the rectal circumference. After the procedure, the patient was submitted to a planning computed tomography scan and the prescribed dose was 30 Gy given in 6 fractions of 5 Gy twice a day, with a minimum interval of 6 h between the fractions (Figures 1 and 2).

In a follow-up evaluation after 9 months of treatment, we observed total healing of the rectal ulcer. The patient reported no more bleeding or pain and stated that she no longer needed to use analgesics.

At 3 months after the end of treatment, a local exam showed neither ulceration nor acute skin reactions. A control magnetic resonance imaging scan, without contrast enhancement, showed a 1.5-mm area of mucosal enlargement in the irradiated area.

## DISCUSSION

LRRC continues to be a serious problem for patients and medical professionals. A second EBRT or CRT is considered for palliative treatment because it is less invasive. However, the curative efficacy of these approaches is not comparable to that of pre-operative CRT followed by re-resection.

Although surgery has a major impact on local control in the small subset of patients with resectable LRRC, extensive resection has been reported to be associated with high morbidity and mortality^(7)^.

The reported 5-year overall survival after treatment for LRRC with surgery plus intra-operative RT or CRT ranges from 5% to 60%^(8,9)^. Wong et al.^(9)^ evaluated 519 patients with LRRC. The median survival was 14 months, and the median time to local disease progression was 5 months after EBRT. The 5-year survival rate was only 5%.

In terms of palliative or radical nonsurgical treatment, HDR-IBT is the most well-established form of re-irradiation. However, the complexity of the procedure and the need to train staff in how to carry it out make its dissemination difficult and restricted to a small number of centers.

There have been a few studies of re-irradiation with EBRT and with HDR-IBT. Das et al.^(10)^ evaluated 48 patients undergoing re-treatment for LRRC with hyperfractionated accelerated EBRT. Patients were treated with 150-cGy fractions twice daily, in a total dose of 39 Gy. Concurrent chemotherapy was administered to all patients. The authors reported a 3-year rate of grade 3 and 4 late toxicity with freedom from local progression in 35% and 47%, respectively. They also observed that the 3-year overall survival rate was 53% in patients with a re-treatment interval of more than 2 years, compared with 21% for those with a re-treatment interval of less than 2 years (*p* = 0.001).

In another study of salvage EBRT, Koom et al.^(11)^ evaluated 22 patients with LRRC treated with re-irradiation of the pelvis. Of those 22 patients, 2 (9%) had grade 3 acute toxicity and 8 (36%) had grade 3-4 late toxicity. The authors also noted that the location of the tumor recurrence (axial or anterior) and surgical resection after re-irradiation significantly influenced severe late toxicity (*p* = 0.024 and *p* = 0.039, respectively) and that re-irradiation doses exceeding 50 Gy significantly increased the in-field progression-free survival (*p* = 0.005)^(12)^.

Morimoto et al.^(12)^ published results related to nine patients with LRRC treated with salvage HDR-IBT. The median age of the patients was 63 years, and the maximum LRRC diameter was 40 mm (range, 20-80 mm). Adenocarcinomas were confirmed histologically in all cases. Four of the nine patients received EBRT, at doses ranging from 21.6 Gy in 12 fractions to 50.4 Gy in 28 fractions, combined with HDR-IBT, with prescribed doses of 30-50 Gy given in 5-10 fractions. Treatment time varied between 3 and 6 days. Five patients were treated with HDR-IBT as salvage monotherapy, receiving 54-60 Gy given in 9-10 fractions over 6 days. With a median follow up of 90 months (range, 6-221 months), local control was achieved in five of the nine patients. The 8-year overall survival, local control, and progression-free survival rates were 56%, 44%, and 33%, respectively. Grade 3 side effects were observed in three patients. Late complications included skin ulceration, vaginal perforation, and vesicovaginal fistula.

Sakurai et al.^(13)^ reported that local control was achieved in seven of 18 patients with LRRC treated with HDR-IBT (30-50 Gy given in 6-10 fractions) and followed for a median of 14.4 months. Tumor progression occurred in 11 patients at a median of 11.5 months after HDR-IBT. No gastrointestinal or urinary complications were observed. No acute Radiation Therapy Oncology Group grade 3 or 4 skin complication was observed, although three patients developed a chronic skin ulcer. Despite the relatively short follow-up periods, the authors demonstrated the potential efficacy of HDR-IBT for the treatment of LRRC. Their long term follow-up report is awaited.

Goes et al.^(14)^ evaluated combined treatment for LRRC, with surgical debulking and intra-operative HDR-IBT or ^125^I, in 30 patients between 28 and 74 years of age. In the patients with gross residual disease, the mean follow-up was 26.5 months, compared with 34.0 months for those with microscopic residual disease, local control being achieved in 37.5% and 66.0%, respectively. At the time of the last follow-up evaluation, the LRRC was under control in 18 patients (64%), 7 patients (25%) showing no evidence of local or distant recurrence.

Kolotas et al.^(15)^ performed 44 HDR-IBT implants in 38 patients with LRRC. Doses ranged from 10-15 Gy given in a single fraction to 30-40 Gy in 5 Gy fractions. After a median follow-up of 23.4 months, 13 of the patients were alive. The median post-HDR-IBT survival was 15 months, and 18 of the 25 deaths were due to distant metastases. A partial tumor response was observed in six patients. Stable disease and local progression were noted in 28 and four patients, respectively. No acute complications were observed. One patient developed a fistula after 8 months. Pain relief was recorded in 34 patients (89.5%). The median duration of the palliative effect was 5 months (range, 1-13 months).

When using HDR-IBT, patient selection is an important factor for demonstrating therapeutic gain. Reports show that there is a relative small chance of gastrointestinal or urinary complications. The chance of developing chronic skin ulcers or fistulas is related to irradiation of the skin or other tissues involved. We recommend that this technique not be used in patients with recurrent tumors involving the skin, vagina, bladder, or an extensive area of the small bowel.

Because of poor perfusion in the tumor bed, LRRC is potentially radio-resistant. The relatively small number of published studies and the heterogeneity of the doses prescribed make it difficult to define an optimal total dose and fractionation schedule. Dose escalation studies are needed in order to develop this approach.

In conclusion, HDR-IBT appears to offer a therapeutic alternative to patients who are not candidates for radical resection or intra-operative RT.

## References

[1] Kapiteijn E, Marijnen CA, Nagtegaal ID (2001). Preoperative radiotherapy combined with total mesorectal excision
for resectable rectal cancer. N Engl J Med.

[2] Bouchard P, Efron J (2010). Management of recurrent rectal cancer. Ann Surg Oncol.

[3] Dent OF, Haboubi N, Chapuis PH (2007). Assessing the evidence for an association between circumferential
tumour clearance and local recurrence after resection of rectal
cancer. Colorectal Dis.

[4] Birbeck KF, Macklin CP, Tiffin NJ (2002). Rates of circumferential resection margin involvement vary
between surgeons and predict outcomes in rectal cancer
surgery. Ann Surg.

[5] Nagtegaal ID, Marijnen CA, Kranenbarg EK (2002). Circumferential margin involvement is still an important
predictor of local recurrence in rectal carcinoma: not one millimeter but
two millimeters is the limit. Am J Surg Pathol.

[6] Magrini S, Nelson H, Gunderson LL (1996). Sacropelvic resection and intraoperative electron irradiation in
the management of recurrent anorectal cancer. Dis Colon Rectum.

[7] Lee JH, Kim DY, Kim SY (2011). Clinical outcomes of chemoradiotherapy for locally recurrent
rectal cancer. Radiat Oncol.

[8] Sun DS, Zhang JD, Li L (2012). Accelerated hyperfractionation fieldinvolved re-irradiation
combined with concurrent capecitabine chemotherapy for locally recurrent and
irresectable rectal cancer. Br J Radiol.

[9] Wong CS, Cummings BJ, Brierley JD (1998). Treatment of locally recurrent rectal carcinoma - results and
prognostic factors. Int J Radiat Oncol Biol Phys.

[10] Das P, Delclos ME, Skibber JM (2010). Hyperfractionated accelerated radiotherapy for rectal cancer in
patients with prior pelvic irradiation. Int J Radiat Oncol Biol Phys.

[11] Koom WS, Choi Y, Shim SJ (2012). Reirradiation to the pelvis for recurrent rectal
cancer. J Surg Oncol.

[12] Morimoto M, Isohashi F, Yoshioka Y (2014). Salvage high-dose-rate interstitial brachytherapy for locally
recurrent rectal câncer: longterm follow-up results. Int J Clin Oncol.

[13] Sakurai H, Mitsuhashi N, Harashima K (2004). CT-fluoroscopy guided interstitial brachytherapy with image-based
treatment planning for unresectable locally recurrent rectal
carcinoma. Brachytherapy.

[14] Goes RN, Beart RW, Simons AJ (1997). Use of brachytherapy in management of locally recurrent rectal
cancer. Dis Colon Rectum.

[15] Kolotas C, Röddiger S, Strassmann G (2003). Palliative interstitial HDR brachytherapy for recurrent rectal
câncer. Implantation techniques and results. Strahlenther Onkol.

